# The impact of sphingomyelin and cholesterol on ordered lipid domain formation in the bovine milk fat globule membrane using artificial giant unilamellar vesicles as a model

**DOI:** 10.3168/jdsc.2024-0719

**Published:** 2025-06-03

**Authors:** Haotian Zheng, Rafael Jiménez-Flores, David W. Everett

**Affiliations:** 1 Dairy Products Technology Center, California Polytechnic State University, San Luis Obispo, CA 93407; 2 Department of Food Science, University of Otago, Dunedin 9016, New Zealand; 3 Department of Food, Bioprocessing and Nutrition Sciences, North Carolina State University, Raleigh, NC 27695; 4 Department of Food Science and Technology, The Ohio State University, Columbus, OH 43210; 5 Riddet Institute, Massey University, Palmerston North 4410, New Zealand

## Abstract

Giant unilamellar vesicle (GUV) bilayers were constructed from polar lipids and cholesterol by electroformation as a model system to investigate the formation of ordered lipid domains (OLD) within the milk fat globule membrane (MFGM). Dark regions without fluorescent staining on the surfaces of GUV, observed by confocal laser scanning microscopy, were characterized as OLD. Lipid formulations were designed by mixing 1,2-dipalmitoyl-*sn*-glycero-3-phosphocholine; 1,2-dioleoyl-*sn*-glycero-3-phosphoethanolamine; milk sphingomyelin; and cholesterol with designated molar ratios to reveal the key components responsible for segregated OLD formation. Cholesterol, rather than milk sphingomyelin, was more responsible for OLD formation. Dark regions were observed in GUV, which contained sphingomyelin but no cholesterol. This observation revealed that at room temperature (below the melting transition temperature [*T_m_*]), not only do cholesterol-mediated ordered domains contribute to lipid phase separation, but phospholipids with high *T_m_* in MFGM are also segregated from the bright fluorescent liquid-disordered domains. This work provides visible evidence demonstrating the comparative roles of sphingomyelin and cholesterol in forming OLD in phospholipid bilayers.

## Highlights

• We used GUV to study the structural nature of milk fat globule membrane.
• The GUV were made by polar lipids and cholesterol relevant to milk fat globule membrane.
• Compared with SM, cholesterol appeared more responsible for OLD formation.

## Graphical Abstract

**Figure:**
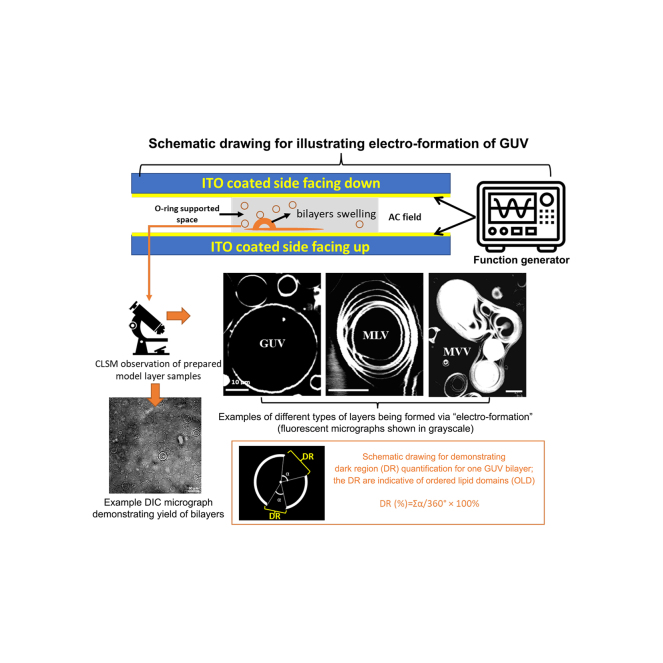
**Summary:** Giant unilamellar vesicles (GUV) were constructed by an electroformation method. Solutions of lipid mixture containing a blend of phospholipids and different contents of cholesterol or milk sphingomyelin (SM) were deposited on the surface of indium tin oxide (ITO)-coated glass slides to form a dried thin film of lipid mixture. The dried layer of lipid mixture was then hydrated by sucrose solution. An alternating current (AC) field was applied to the small sample reservoir within an O-ring space. The applied electrical field stimulated a swelling effect of the deposited lipid layers. Consequently, lipid layers constructed by the mixed lipidic components were formed; these systems include GUV, multilamellar vesicles (MLV), and multivesicular vesicles (MVV). By analyzing the (nonfluorescent) dark regions in GUV constructed by different lipid mixtures containing different amounts of SM and cholesterol, it was suggested that cholesterol might be an effective component in determining ordered lipid domain (OLD) formation. CLSM = confocal laser scanning microscopy; DIC = differential interfacial contrast.

Fat is present in fresh bovine milk as natural globular emulsion oil droplets, namely bovine milk fat globules (**MFG**), with size ranging from 200 nm to >15 µm (Lopez, 2020). Milk fat globules are encapsulated by the milk fat globule membrane (**MFGM**) which has a thickness of ∼10 to 20 nm (Mather, 2011). This membrane consists of a complex arrangement of polar lipids (phospholipids and sphingolipids) and cholesterol forming a trilayer membrane framework (Zheng et al., 2014a). Modulating the interfacial structure of bioinspired emulsion oil droplets using MFGM lipids for novel nutrition formula applications has gained great interest in recent years (Gallier et al., 2015; Lopez et al., 2019; Ma et al., 2023; Ma et al., 2024). Segregated lipid domains can be seen on the surface of native MFG using confocal laser scanning microscopy (**CLSM**) coupled with a fluorescent staining method. The lateral segregation of lipid domains in MFGM was revealed by contrasting the “bright” fluorescent and the “dark” nonfluorescent regions on the surfaces of MFG (Gallier et al., 2010; Lopez et al., 2010). The dark regions were interpreted as lipid rafts, the liquid-ordered domains (***L*_o_**) rich in milk sphingomyelin (**SM**) and cholesterol; these regions were surrounded by the bright regions, the liquid-disordered domain (***L*_d_**) composed of other glycerophospholipids. A schematic drawing is shown in Figure 1 to demonstrate the fluorescent bright regions and the nonfluorescent dark regions in a model bilayer. In a model phospholipid bilayer system, ordered lipid domains (**OLD**) may be a solid gel phase (***L*_β_**) as composed by polar lipids with high melting transition temperatures (***T_m_***) or an *L*_o_ phase composed by polar lipids and cholesterol; the OLD may induce mechanical heterogeneities in MFGM, which might be impactful regarding the adsorption and entering behavior of digestive enzymes toward MFGM (Lopez et al., 2019). Because the gastrointestinal digestion of oil droplets is governed by an interfacial process, including adsorption of digestive enzymes (McClements, 2021). Understanding the formation mechanism of OLD and their functionality is essential for a rational design of interfacial composition or structure of processed emulsion oil droplets for nutrition formula applications. The roles of milk SM and cholesterol on OLD formation and functionality have been investigated and revealed using “flat” supported lipid bilayer (**SLB**) model systems (Lopez et al., 2019; Obeid et al., 2019). Zheng et al. (2014b) constructed spherical-like giant unilamellar vesicles (**GUV**) as an artificial model system mimicking some characteristics of the surface morphology of MFGM. The GUV in the referenced work were made using selected polar lipids according to MFGM composition, and the authors found that the segregated dark phases, *L*_o_, in GUV surfaces may be due to asymmetric distribution of 1,2-dipalmitoyl-*sn*-glycero-3-phosphocholine (16:0-phosphatidylcholine, **DPPC**) and 1,2-dioleoyl-*sn*-glycero-3-phosphoethanolamine (18:1-phosphatidylethanolamine, **DOPE**) between the 2 leaflets of the lipid bilayer as observed under CLSM at ambient temperature.

**Figure 1:**
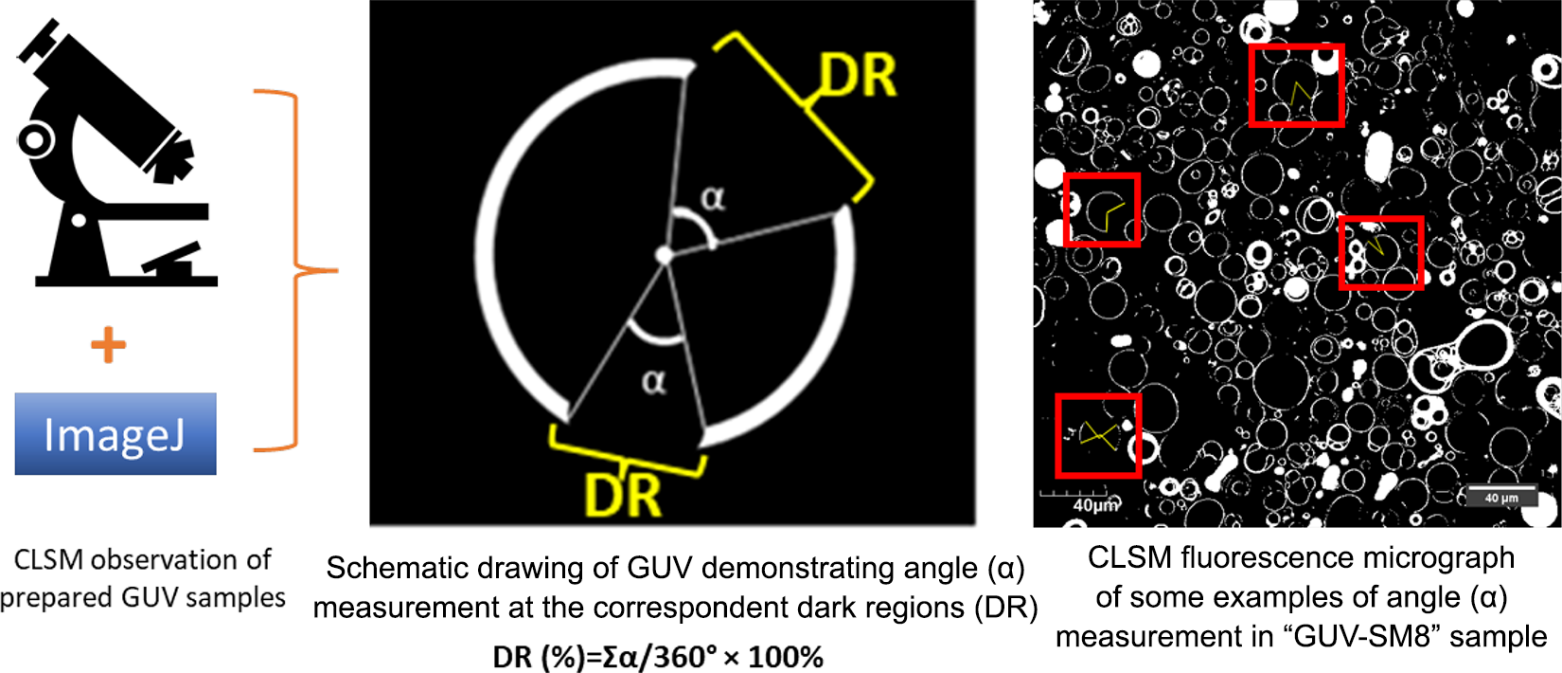
Schematic illustrations of the quantification approach of the relative ratio of nonfluorescent dark regions (DR, %) in GUV.

Considering these previous findings, we hypothesize that it is technologically possible to conduct a study to quantitively demonstrate how milk SM and cholesterol modulate OLD formation using the spherical GUV bilayers consisting of major polar lipids found in MFGM. To investigate this further, in the present work, GUV were constructed from designated combinations of lipids containing both polar lipids and cholesterol. The formation of OLD within the GUV as model of the MFGM was examined to further understand the role of milk SM and cholesterol on lateral lipid domain segregation.

Giant unilamellar vesicles were constructed from designed lipid mixtures by electroformation based on previous studies (Herold et al., 2012; Zheng et al., 2014b) with modifications. Giant unilamellar vesicles were formed in a 100 m*M* sucrose solution in a custom designed sample chamber, and an alternating current (3 V peak-peak, 10 Hz) was applied at 55 ± 1°C for 45 min on the surface of the indium tin oxide slides for producing lipid bilayers through swelling (Morales-Penningston et al., 2010). A schematic illustration of GUV formation is shown in the graphical abstract; GUV, multilamellar vesicles (**MLV**), and multivesicular vesicles (**MVV**) may be found in the prepared model layer samples. Multilamellar vesicles and MVV were identified based on schematic morphological features as illustrated in literature (Milcovich et al., 2017). In the present study, only GUV were selected for analysis.

Giant unilamellar vesicles were formed from ternary or quaternary systems comprising DPPC, DOPE, and SM, without or with cholesterol. A control GUV sample that contains DPPC, DOPE, SM and cholesterol with a relative molar ratio of 8:8:8:4 was prepared; this relative molar ratio is equivalent to a percentage molar ratio of 29:29:29:14 mol%. The relative molar ratio design principle for the control sample is analogous to previous work regarding MFGM morphology investigation, in which the authors prepared MLV using SM, DOPC, and cholesterol with a percentage molar ratio of 40:40:20 mol% (Guyomarc'h et al., 2014). In the referenced work, the molar ratio between polar lipids (e.g., SM/DOPC) was 1:1 and the molar ratio between a polar lipid and cholesterol was 2:1. The control GUV sample was compared with MFG from raw milk regarding the observed nonfluorescent regions on their surfaces; example CLSM fluorescent micrographs of control GUV and MFG are shown in Figure 2A. The relative molar ratio of control sample is used as a baseline, upon which the relative molar ratios of SM and cholesterol were altered to investigate their impact on OLD formation in the GUV.

**Figure 2:**
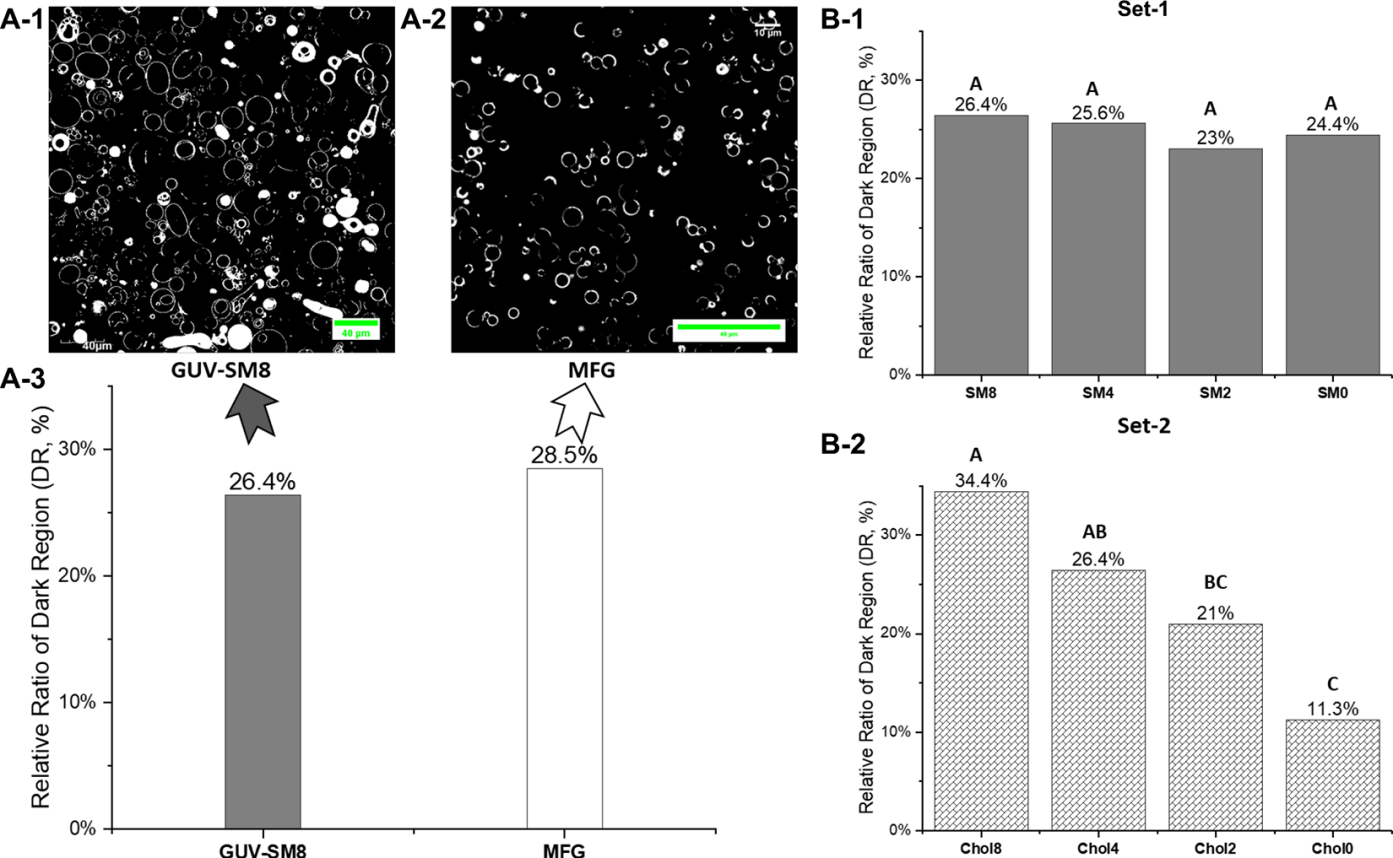
(A) A-1 and A-2: Grayscale CLSM fluorescent micrographs of corresponding samples, control GUV and MFG from raw milk. A-3: Estimated proportions of dark regions in the surfaces of GUV and MFG. (B) Estimated proportions of dark regions plotted as a function of altered relative molar ratios of SM and cholesterol, respectively. B-1 and B-2 are for set-1 and set-2 samples, respectively. Means that do not share an identical uppercase letter shown on top of individual bars suggest statistical significance (*P* < 0.05).

Briefly, 2 sets of recombined lipid mixture samples (set-1 and set-2, shown in Figure 2B) were prepared by manipulating relative molar ratio levels of SM (in set-1) and cholesterol (in set-2), while the relative molar proportions of other lipid components were kept unchanged. Set-1 had 4 samples, which contained an identical relative molar ratio of DPPC, DOPE, and cholesterol (8:8:4), and the SM contribution to the relative molar ratio was manipulated ranging from 8 to 0; the sample names are referred as SM8 to SM0, as shown in Figure 2B**-**1. Similarly, set-2 also had 4 samples, in which the relative molar ratio for DPPC, DOPE, and SM was identical (8:8:8). The cholesterol contribution to the relative molar ratio was altered from 8 to 0; the sample names are referred as Chol8 to Chol0, as shown in Figure 2B**-**2. The equivalent molar percentage ranges for SM and cholesterol in the recombined lipid mixtures were estimated as 0% to 29 mol% and 0% to 25 mol%, respectively. The molar masses of DOPE (744.034 g/mol), DPPC (734.039 g/mol), milk SM (785.034 g/mol), and cholesterol (386.7 g/mol) were used for calculating the required weights of individual lipid ingredients for sample preparation. Mixed lipid ingredients were dissolved in chloroform to yield ∼10 mg/mL sample solutions, which were premixed with a fluorescent stain (0.25 mol %), 1,2-dioleoyl-*sn*-glycero-3-phosphoethanolamine-N-(lissamine rhodamine B sulfonyl) (ammonium salt) (**Rd-DOPE**), for GUV construction. It has been discussed that Rd-DOPE may intercalate into the looser *L_d_* phase in GUV, leaving the more compacted OLD composed by *L_o_* and *L_β_* phases nonfluorescent and dark in appearance under CLSM imaging (Zheng et al., 2014b). Therefore, the impacts of SM and cholesterol on OLD formation may be investigated by quantifying the dark nonfluorescent regions in GUV constructed by lipid mixtures from set-1 and set-2. In general, dye concentrations lower than 1 mol% do not affect membrane physical properties (Bouvrais et al., 2010).

An inverted CLSM system (Olympus FV1000, Olympus America Inc., Center Valley, PA) with a 40× oil-immersion objective lens was used to observe GUV. The Rd-DOPE-labeled bilayers were excited by a diode laser (559 nm), and the emitted fluorescent light was collected between 570 and 670 nm. Example GUV produced by electroformation are shown in a differential interfacial contrast micrograph along with fluorescent micrographs of GUV and giant multilamellar vesicles (**GMV**) in the graphical abstract. The CLSM observations were carried out at an ambient temperature (22 ± 1°C). No fewer than 9 CLSM micrographs were taken for each GUV formula (note that the lipid composition for samples SM8, Chol4, and the control sample [GUV-SM8] shown in Figure 2 are the same sample). The angles formed by dark regions (shown as α in the schematic demonstration in Figure 1) were measured by using the ROI (region of interest) function in the ImageJ software (version 1.51J8, National Institutes of Health, Bethesda, MD). The proportion of nonfluorescent dark regions of individual GUV (n = 15) was calculated from the ratio of the summation of angle (Σα) for each dark region on a 2-dimensional circle, and 360° representing the full circle angle; a schematic illustration of GUV formation and is shown in Figure 1. The proportion of dark regions (**DR**, as a percentage) can therefore be calculated from the following equation:

$$DR\left(\%\right)=\frac{\Sigma \alpha }{360^{\circ }}\times 100\%.$$

A 2-sample t-test and 1-way ANOVA with Fisher LSD test were performed using OriginPro 2024b (64-bit, Academic, OriginLab Corporation, Northampton, MA) to reveal significance among sample means; the significance level was set at 0.05.

Model lipid bilayers from the control sample swelled and formed circular shapes under the electroformation conditions. Bilayers were generated as circular shapes with a typical GUV structure and irregular shapes of bilayer complexes referred as GMV (see the graphical abstract for example GUV and GMV); these were also observed in previous reports (Estes and Mayer, 2005; Politano et al., 2010). Only circular-shaped GUV were used for further characterization.

Dark regions in the surfaces of GUV and MFG, as seen by CLSM, are characterized as OLDs (Lopez et al., 2010; Zheng et al., 2013, 2014b). No statistical difference was observed in the proportion of DR (%) between the control GUV sample and MFG from raw milk (Figure 2A), suggesting that the control GUV sample, which is identical to samples of SM8 and Chol4, can represent the characteristic phenomena of OLD segregation from the disordered domains in the surfaces of MFG sourced from raw milk.

In the quaternary and ternary systems including samples from set-1 and set-2, the relative amounts of DR in GUV surfaces were measured in response to depletion of SM or cholesterol, respectively. Depleting SM concentration had no effect on the relative proportion of observed DR (Figure 2B-1), whereas the formulas with cholesterol depletion resulted in GUV with a reduction in the DR in the surface (Figure 2B-2). Although the Chol8 sample showed DR accounting for ∼30% of the total surface area on average (Figure 2B-2), some of the GUV showed no observable DR; examples are shown in Figure 3A. This observation was unexpected. A possible explanation of these unexpected observations is that heterogeneous distributions of polar lipids and cholesterol in different GUV may cause insufficient presence of cholesterol and high *T_m_* polar lipids in some of the GUV; therefore, an OLD is not able to be formed, or any ordered domains that are formed are smaller than the resolution of the microscope.

**Figure 3:**
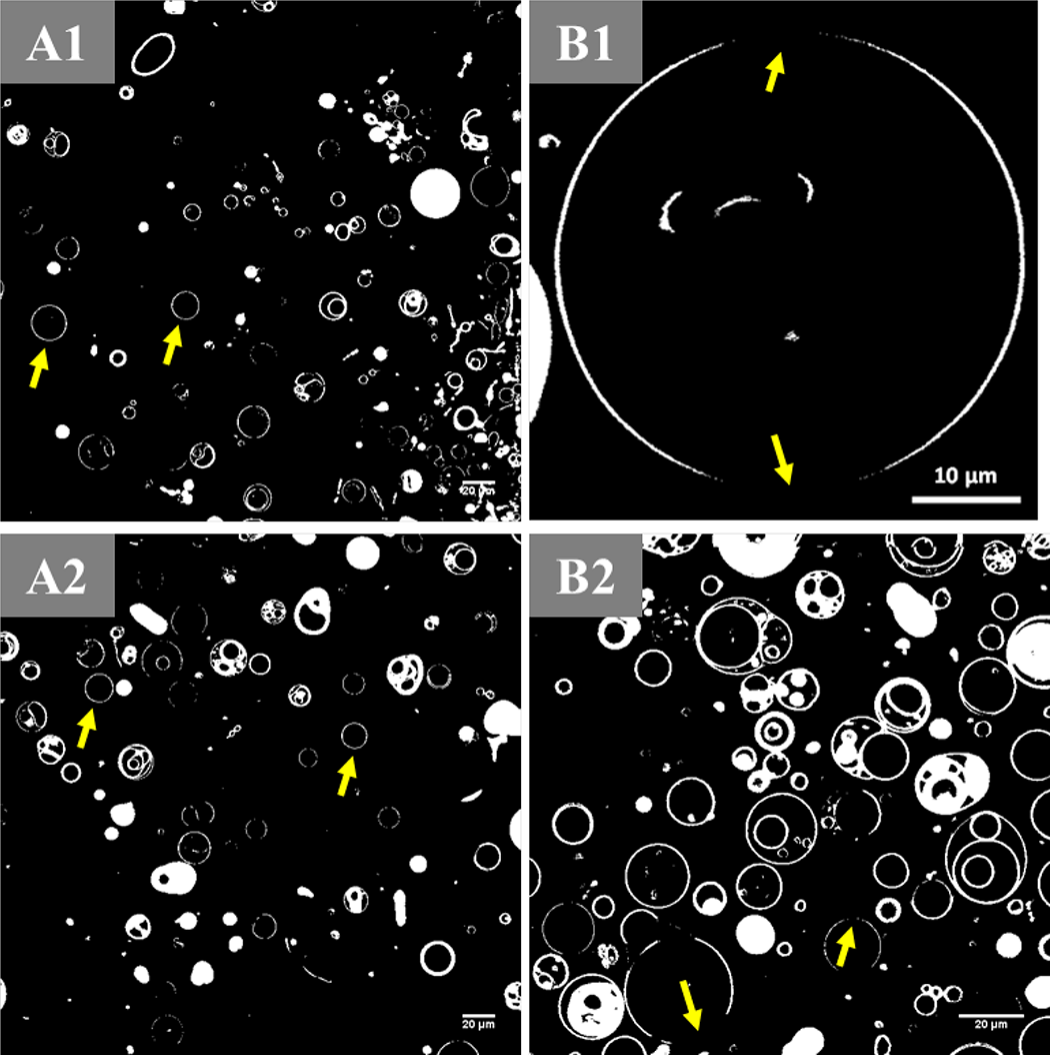
Examples of CLSM fluorescent micrographs of GUV. A1 and A2: Sample Chol8 containing DPPC, DOPE, SM, and cholesterol with a relative molar ratio of 8/8/8/8. Arrows point to GUV without observable OLD. B1 and B2: Sample Chol0 made from a lipid mixture with a relative molar ratio of 8/8/8/0 for the components of DPPC, DOPE, SM, and cholesterol, respectively. Arrows point to nonfluorescent regions, which are indicative of OLD.

Moreover, the segregated nonfluorescent dark domains were observed in GUV made from sample Chol0 without the inclusion of cholesterol (Figure 3B). This result suggests that at room temperature, although *L_o_* domain in the MFGM is rich in cholesterol as discussed in the literature (Lopez, 2010), cholesterol might not be a requisite component to cause a lipid phase separation in the GUV system composed of MFGM polar lipids due to a possible formation of a solid-like gel phase (*L_β_*). For instance, Murthy et al. (2016) studied the topography of “flat” SLB constructed by MFGM lipids and reported an observation of gel phase domain segregated from the *L_d_* domain in in a model SLB, in which cholesterol was absent.

Lopez et al. (2018) characterized thermotropic phase behaviors of SM and SM-cholesterol mixtures and found that the phase transition temperature of hydrated milk SM bilayers was 34.3 ± 0.1 °C. These authors also provided evidence that an SM-cholesterol–induced *L_o_* phase is temperature independent within the range of tested temperatures (10–60°C) crossing the *T_m_* of SM. In the current work, GUV were formed at a temperature above the *T_m_* of SM but were observed and characterized at room temperature (<*T_m_* of SM); it is therefore reasonable to speculate that a presence of SM-induced gel phase (*L_β_*) in the GUV sample of Chol0 may be a possibility.

Milk SM may not necessarily be the only polar lipid, in association with cholesterol, responsible for the *L_o_* phase formation in the MFGM or in the GUV surfaces made from polar lipids of MFGM (Zheng et al., 2014a,b). The competitive affinity of cholesterol for glycerophospholipids and sphingophospholipids in the formation of lipid domains has been reviewed by McMullen et al. (2004), and interactions between glycerophospholipids and cholesterol in the presence of SM were described for human erythrocyte membrane, a system that is analogous to the MFGM, which also contains phosphatidylcholine, phosphatidylethanolamine, phosphatidylserine, and SM. McMullen et al. (2004) pointed out that although cholesterol has a higher affinity to SM compared with glycerophospholipids, a significant amount of cholesterol may be found in the glycerophospholipid phase, which was immiscible to the SM phase. These authors speculated that glycerophospholipid domains relatively depleted of cholesterol and SM domains enriched with cholesterol would both exist in the *L_o_* phase. This may be interpreted as indicating that SM is not a critical factor in determining the appearance of OLD where glycerophospholipids and cholesterol coexist. Figure 2B-1 provided indirect evidence for this speculation, showing that depletion of SM relative molar ratio in the model lipid mixture did not alter the relative quantity of DR. A deduced mechanism may be explained as follows: upon depleting milk SM in GUV samples in set-1, the cholesterol may still be able to interact with the saturated phospholipid, DPPC, to form OLD. This mechanism is in a good agreement with a previously deduced schematic model of MFGM, which showed that cholesterol interacts with acyl chains of DPPC and formed a *L_o_* phase (Zheng et al., 2014a). Moreover, a relatively high association constant for DPPC and cholesterol in the *L_o_* phase was reported previously (Yang et al., 2016). Therefore, with the presence of various types of glycerophospholipids, the molar proportion of milk SM seemed not to be a major factor in regulating *L_o_* phases or OLD, as indicated by the estimated quantities of DR in GUV.

Using monolayer (Langmuir–Blodgett) model films (Murthy et al., 2015) and MLV (Murthy et al., 2016) constructed by MFGM polar lipids, a condensing effect of cholesterol in the mono- and bilayer systems with the co-presence of MFGM polar lipids was found. These authors also showed that cholesterol was able to modulate (1) the geometry of segregated lipid domains and (2) the surface pressure–area (π-A) compression isotherms. These tunable surface properties, as modulated by lateral interactions between polar lipids and cholesterol, could influence the digestion fate of emulsified oil droplets in a processed nutrition formula. This hypothesis is worthy of future study. In the current work, we provided additional indirect evidence of the cholesterol condensing effect using engineered GUV bilayer systems. The changing trend of dark region quantity of GUV made from the set-1 samples with a depletion of SM shown in Figure 2B-1 suggests that cholesterol was incorporated into the DPPC/DOPE bilayers, thus forming OLD, the nonfluorescent regions. These findings provide further information on the functionality of cholesterol on OLD formation to what has already been reported and discussed by (Murthy et al., 2015, 2016; Lopez et al., 2018).

The primary novelty of this work was in preparing manufactured spherical-like unilamellar bilayers to demonstrate the role of cholesterol and milk SM in forming lateral OLD within a MFGM. This work demonstrates that cholesterol, rather than milk SM, played a more substantial role in regulating the formation of OLD in a system that mimics the bovine MFGM. The results may serve as a guidance for food structure design of nutrition formulas in the future when mimicking the surface structure of MFG is of interest.
